# eRNAs and Superenhancer lncRNAs Are Functional in Human Prostate Cancer

**DOI:** 10.1155/2020/8847986

**Published:** 2020-09-22

**Authors:** Xiaona Zhang, Panpan Pang, Min Jiang, Qunfa Cao, Huili Li, Yi Xu, Yao Li, Xue Chen, Junsong Han

**Affiliations:** ^1^Department of Pathology, Tongji Hospital, Tongji University School of Medicine, Shanghai, China; ^2^National Engineering Center for Biochip at Shanghai, Shanghai, China; ^3^Shanghai Biochip Corporation, Shanghai, China; ^4^Shanghai Engineering Research Center of Industrial Microorganisms, School of Life Science, Fudan University, Shanghai, China; ^5^Yueyang Hospital of Integrated Traditional Chinese and Western Medicine, Shanghai University of Chinese Medicine, Shanghai, China

## Abstract

Prostate cancer (PCa) is one of the most commonly diagnosed cancers in males worldwide. lncRNAs (long noncoding RNAs) play a significant role in the occurrence and development of PCa. eRNAs (enhancer RNAs) and SE-lncRNAs (superenhancer lncRNAs) are important elements of lncRNAs, but the role of eRNAs and SE-lncRNAs in PCa remains largely unclear. In this work, we identified 681 eRNAs and 292 SE-lncRNAs that were expressed differentially in PCa using a microarray. We also found that eRNAs transcribed from active open chromatin had significantly higher expression than those from active closed chromatin, and SE-lncRNAs had a little higher expression than eRNAs. Next, we constructed a transcriptional regulation network that eRNA-related enhancer and the target genes shared the same TF-binding motifs. Further, we investigated whether CTCF played a role in mediating the transcriptional regulation network. eRNAs, especially those that regulate androgen response genes, may be candidates for prognostic biomarkers and therapy targets. Our work provides a new perspective for developing medical treatments and therapies for prostate cancer.

## 1. Introduction

In the last decade, it has been shown repeatedly that the genomes of many species are transcribed pervasively to produce noncoding transcripts [1]. A class of noncoding RNAs, typically more than 200 nucleotides, is referred to as long noncoding RNA (lncRNA). lncRNAs have been proposed to carry out diverse functions that include transcriptional regulation at multiple levels, organization of nuclear domains, and regulation of proteins or RNA molecules [2–5]. According to the characteristics of lncRNA transcripts, there are several types of lncRNAs, which include intergenic transcripts, sense or antisense transcripts that overlap other coding genes, and eRNAs [6]. A novel class of enhancer transcribed lncRNAs, referred to as enhancer RNA (eRNA) [7–11].

Enhancers [12] are key *cis*-acting gene regulatory elements in eukaryotes, which can effectively promote the expression of target genes [13]. They can sustain precise control of transcription by serving as binding platforms for transcription factors (TFs) and cofactors that regulate productive transcription at core promoters during development; their misregulation contributes to human diseases [14–16]. Enhancers are generally found within accessible chromatin [14–16]. Nevertheless, they can either be located close to the core promoter or distal to their target genes [17–19]. Genome-wide analysis suggests that enhancers may be transcribed generally [7, 20]. eRNAs are expressed in a tissue-specific manner [21, 22], and they play an important role in enhancer activity and regulating gene transcription by diverse mechanisms [23–28]. eRNAs are involved in the enhancement of transcription. For instance, eRNA production from p53-bound enhancer regions (p53BERs) was required for efficient enhancement of p53 transcription [24]. Nuclear receptors, such as estrogen receptor (ER), androgen receptor (AR), and Rev-erbs-related eRNAs, have been demonstrated that eRNA knockdown can lead to correlate changes in target gene expression [23, 25, 29–31].

Superenhancers (SE) consist of large clusters of transcriptional enhancers that are distinct from the typical enhancers in their ability to activate cell-type and tissue-specific genes and result in a higher susceptibility for disease. SE-lncRNA CCAT1-L interacted with CTCF and modulated chromatin conformation at the MYC loop regions in colorectal cancer [32]. SE-lncRNA UCA1 enhanced the AMOT-YAP interaction to promote YAP dephosphorylation and nuclear translocation to activate YAP target genes in epithelial ovarian cancer [33].

Prostate cancer (PCa) is one of the most commonly diagnosed cancers in males worldwide [34, 35], and it remains the third most common killer in men with cancer [36]. The role of eRNAs and SE-lncRNAs in prostate cancer remains largely unclear. In this study, our custom-designed noncoding RNA microarrays were used to compare the expression profiling of prostate tumors and corresponding adjacent normal prostate tissues to identify the differentially expressed eRNAs and SE-lncRNAs and to explore their regulated functions.

## 2. Materials and Methods

### 2.1. Sample Preparation

Tumor and corresponding adjacent normal prostate tissues were obtained from three PCa patients at the Fudan University Shanghai Cancer Center. Those patients did not receive any preoperation treatment. The study was approved by the Research Ethics Committee of Fudan University Shanghai Cancer Center. Informed consent was provided by all patients. All samples were collected and used for gene expression analysis by microarray and qRT-PCR. Total RNA was isolated from tumor and corresponding adjacent normal prostate tissues using TRIzol Reagent (Life Technologies, Carlsbad, CA, US), according to the manufacturer's instructions. All samples were checked for a RIN number to inspect RNA integration by an Agilent Bioanalyzer 4200 (Agilent Technologies, Santa Clara, CA, US).

### 2.2. Microarray Design

A custom microarray was designed by eArray, a web-based application for Agilent's custom microarray design from Agilent Company. The microarray contains 93221 lncRNAs and 27482 mRNAs; the probe length is 60 nt. The entire set of protein-coding mRNA from Refseq of NCBI and lncRNA contents are based on the database of NCBI, UCSC, Ensembl, GENCODE, LNCipedia, lncRNAdb, and NONCODE.

### 2.3. Microarray Assay and Data Analysis

Qualified total RNA was further purified by a RNeasy microkit (Cat#74004, QIAGEN, GmBH, Germany) and a RNase-Free DNase Set (QIAGEN, GmBH, Germany). Purified RNA was then used to generate fluorescence-labeled cRNA for the SBC human ceRNA array (4 × 180 K). The hybridization solution was prepared according to the *in situ* hybridization kit plus (Agilent Technologies, Santa Clara, CA, US). Hybridization was carried out using a custom microarray at 60°C for 18 h. After hybridization, the slide was scanned on an Agilent Microarray Scanner (Agilent Technologies, Santa Clara, CA, US). Data were extracted with Feature Extraction software 12.1 (Agilent Technologies, Santa Clara, CA, US). The raw data were normalized by the limma package in R software, and the quantile algorithm was used. The normalized signal was calculated by log2.

Ratios were calculated between the two groups using Student's *t*-test and fold change. Hierarchical clustering was applied to elucidate the diacritical eRNA expression pattern. Statistically significant eRNAs that were expressed differentially were displayed using a volcano plot that was filtered by fold change ≥ 2, *p* value < 0.05.

### 2.4. Identification of eRNA and SE-lncRNA

Enhancers were downloaded from three sources, and redundancy was removed: ENCODE DHS (DNase I hypersensitivity sites) data were downloaded from the DENdb database (https://www.cbrc.kaust.edu.sa/dendb/) [37], and H3K27ac CHIP-seq and DNase-seq data were downloaded from ENCODE (https://www.encodeproject.org/) [38]. Then, active enhancers were identified following the rule: active enhancer must be open chromatin and have high H3K27ac max-Zs. If they were TSS proximal, they also had to have low H3K4me3 max-Zs. Furthermore, each enhancer was classified as being proximal (≤2 kb) or distal (>2 kb) to the nearest GENCODE annotated TSS. The third part of active enhancers that was identified by STARR-seq in LNCaP cells was downloaded from GSE82204 (https://www.ncbi.nlm.nih.gov) [14]. STARR-seq is a massively parallel reporter assay to identify active enhancers based directly on their activity in entire genomes, so the enhancers identified by STARR-seq may be in closed chromatin. Superenhancers were downloaded from superdb (https://sourceforge.net/projects/superdb/). eRNAs and SE-lncRNAs were identified from lncRNAs using a SBC human ceRNA array where a minimum of one nucleotide overlapped with above enhancers and superenhancers using BEDTools [39].

### 2.5. Prediction of eRNA Target Genes

Differentially expressed eRNAs were selected for target gene prediction as described previously. The Refseq genes within a 300 kb window upstream or downstream of eRNAs were considered to be target genes using ClosestBed functionality in BEDTools. Corresponding target genes that were expressed differentially in the microarray with eRNA were selected further.

### 2.6. Analysis of Functional Enrichment of Target Genes

To better understand the biological functions and pathways of the differentially expressed target genes, Gene Ontology (GO) was analyzed using the R/Bioconductor package clusterProfiler, and the Kyoto Encyclopedia of Genes and Genomes (KEGG) pathway was analyzed by DAVID (https://david.ncifcrf.gov/). Functional enrichment analysis was based on the threshold of *p* value < 0.05.

### 2.7. TCGA Data Analysis

To validate eRNA microarray results, RNA-seq data that were generated with 33 paired tumors from patients with nonrecurrent prostate cancer and patients with normal tissues were obtained from TCGA (https://tcga-data.nci.nih.gov/tcga/), which was imputed on an Illumina HiSeq RNA-seq platform. Statistical analysis was done using the Mann-Whitney-Wilcoxon test with RPKM for all tumors and normal tissues. The differentially expressed RNAs of the data set were filtered by fold change ≥ 2, *p* value < 0.05. The differentially expressed eRNAs were identified based on the Integrated Annotation of Ensembl (http://asia.ensembl.org/index.html) and microarray eRNAs. RNA-seq data and clinical information of PCa are publicly available and in open access platforms. Therefore, approval by the local ethics committee was not needed.

### 2.8. qRT-PCR Assay

The RNA of each group was reverse transcribed to cDNA with the PrimeScript RT reagent Kit (TaKaRa, Dalian, China). Quantitative real-time PCR assay was performed with SYBR Premix Ex Taq™ II (TaKaRa, Dalian, China) and a 7900 system (Applied Biosystems, Carlsbad, CA, U.S.A.) in triplicate for each sample, according to the manufacturer's instructions. All the primer sequences are listed in Supplementary Table 1 the raw data are listed in Supplementary Table 3. The expressions of specific genes were calculated using the comparative 2^−ΔΔCt^ method with *β*-actin as a calibrator.

### 2.9. Prediction of Transcription Factors (TF) and Construction of Interaction Networks

The prediction of potential TFs aimed at target genes and enhancers was done by HOMER (http://homer.ucsd.edu/homer/) motif analysis for the promoter of eRNA target genes and eRNA-related enhancers. For target genes, the search areas included DNA 2 kb upstream of each gene locus, and the search area for enhancers was its chromatic locus. The interaction of enhancer (eRNA) and target gene promoters that shared the same TF-binding motifs was confirmed by chromatin interaction region data that were generated with 3C, 4C, and 5C and a ChIA-PET assay from DENdb database [37]. The selected enhancer- (eRNA-) TF target gene coordination formulated the interaction networks using Cytoscape 3.4.0 software (Agilent and IBS, Santa Clara, CA, U.S.A.). The degree represented the importance of eRNAs, enhancers, TFs, and target genes in the network.

### 2.10. CTCF ChIP-seq Data Analysis

To determine whether CTCF contributed to the mediation of chromatin looping of enhancer-target gene promoters, CTCF ChIP-seq data in LNCaP cells were downloaded from ENCODE. We then measured whether CTCF enrichment in both enhancers and promoters of 147 eRNA- (enhancer-) TF target genes was coordinated as previously analyzed by BEDTools.

### 2.11. Public Data Download

ENCODE DHS and chromatin interaction region data that were generated with 3C, 4C, and 5C and a ChIA-PET assay were downloaded from the DENdb database (https://www.cbrc.kaust.edu.sa/dendb/). H3K27ac CHIP-seq in the PC-3 cells, CTCF ChIP-seq in LNCaP cells, and DNase-seq data were downloaded from ENCODE (https://www.encodeproject.org/). STARR-seq in LNCaP cells was downloaded from GSE82204 (https://www.ncbi.nlm.nih.gov). Superenhancers were downloaded from superdb (https://sourceforge.net/projects/superdb/). The transcriptome data and corresponding clinical information of 33 nonrecurrent prostate cancer PCa patients were obtained from TCGA data (https://tcga-data.nci.nih.gov/tcga/), which was imputed on the Illumina HiSeq RNA-seq platform.

## 3. Results

### 3.1. Identification of Differentially Expressed eRNAs in Prostate Cancer

We designed a custom microarray containing 93221 lncRNAs and 27482 mRNAs using eArray, a web-based application for Agilent's custom microarray design, namely, SBC human ceRNA array. To identify eRNAs and SE-lncRNAs contained in our custom-designed noncoding RNA microarray, we downloaded enhancers from ENCODE, the DENdb database, and STARR-seq data from the GEO database (GSE82204). DENdb-integrated enhancers were predicted by five different methods (CSI-ANN, Segway, ChromHMM, RFECS, and the ENCODE integrative annotation) that generated an enriched catalogue of putative enhancers for each of the analyzed cell lines [37]. A total of 470,432 ENCODE DHS that supported enhancers were obtained from the DENdb database. The ENCODE (Encyclopedia of DNA Elements) project identified and analyzed all regulatory elements in the human genome [38]. The high-quality histone mark enrichment ChIP-seq and DNase-seq data were downloaded. A total of 55,603 active enhancers were identified from ENCODE data in PC-3 cells. Whole-genome STARR-seq is a method that can obtain active enhancers more reliably. Liu et al. identified 94,527 active enhancers by whole-genome STARR-seq in LNCaP cells [14].

**Figure 1:**
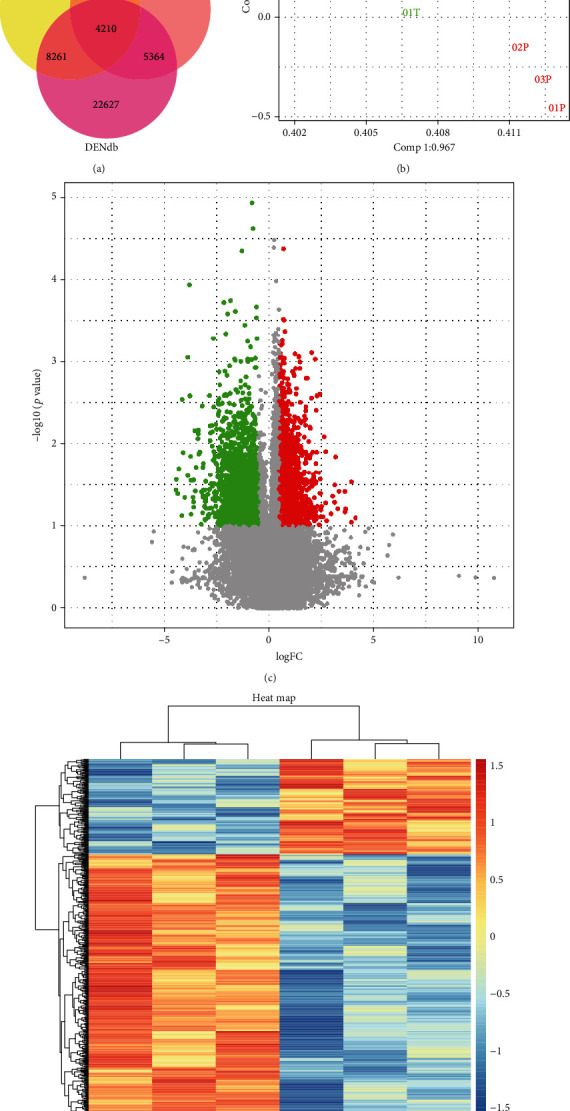
Overview of differentially expressed eRNAs in prostate cancer. (a) Venn diagram showing the overlap of eRNAs identified from DENdb, ENCODE, and STARR-seq (GSE82204). (b) Principal component analysis (PCA) score plots, the expression pattern of each sample from the same condition were similar, which showed the high reliability and reproducibility of the microarray data. (c) Differentially expressed eRNAs in a microarray were filtered by a volcano plot that was filtered by fold change ≥ 2, *p* value < 0.05, where red shows upregulated and green shows downregulated. (d) A heat map of differentially expressed eRNAs in a microarray, where red shows upregulated and blue shows downregulated.

All identified enhancers that overlapped lncRNAs contained in the microarray were further identified as eRNAs [40]. Next, we integrated the three parts of eRNAs after removing redundancy, and 43,680 eRNAs were identified (Figure 1(a); Table 1).

To investigate transcriptional networks of all regulatory elements and to uncover the role of eRNAs and SE-lncRNAs in prostate cancer, we assessed the eRNA and mRNA expression patterns in prostate cancer. Three paired prostate tumors and corresponding adjacent normal tissues were analyzed using the microarray.

**Figure 2:**
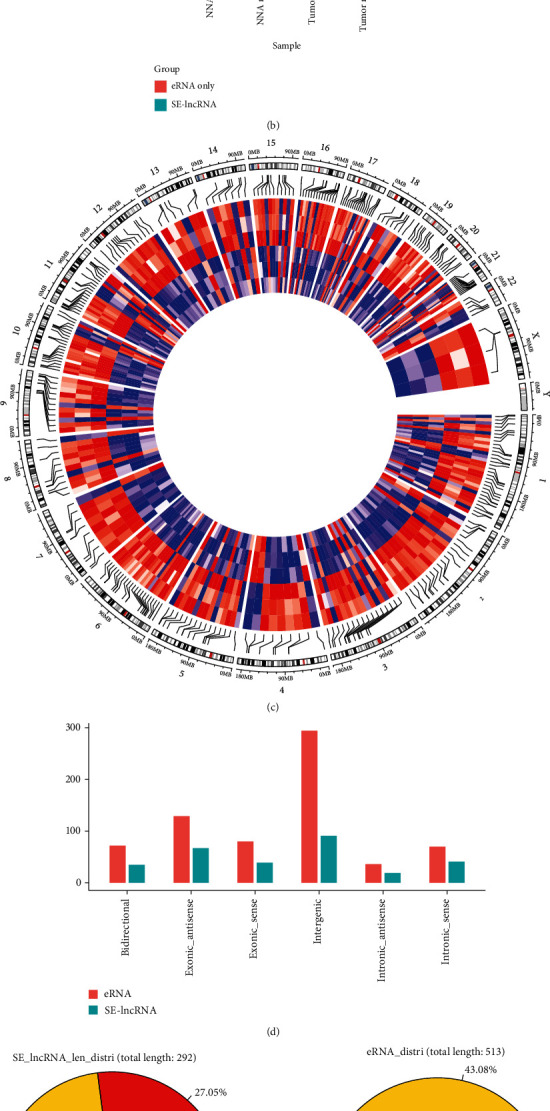
Overview and bioinformatics analysis of eRNAs and SE-lncRNA expression in prostate cancer. (a) Box plot of signals of eRNAs in a microarray that were transcribed from active open chromatin and closed chromatin in LNCaP cells. (b) Barplot of signals of eRNAs and SE-lncRNAs in a microarray. (c) Circos diagram that shows the chromosome distribution of differentially expressed SE-lncRNAs in a microarray. (d–f) The distributions of differentially expressed eRNAs and SE-lncRNAs in a microarray were analyzed based on three criteria: classification (d), length (e), and chromosome locus (f).

For quality control, we compared the expression of all eRNAs and mRNAs obtained from all of the samples by PCA to ensure that variance in the expressions between prostate tumors and corresponding adjacent normal tissues existed, and the conditions were similar (Figure 1(b)). Then, 681 differentially expressed eRNAs and 1220 differentially expressed mRNAs were identified by Student's *t*-test using the threshold of *p* value < 0.05 and fold change ≥ 2 (Figures 1(c) and 1(d); Table 1). The distributions of differentially expressed eRNAs were analyzed based on three criteria: classification, length, and chromosome locus (Figures 2(d)–2(f)). The GEO accession number for the microarray data is GSE155056.

### 3.2. The Expression of eRNAs Transcribed from Active Enhancers

eRNAs that were transcribed from active enhancers were thought to be active eRNAs. We analyzed the expression of active eRNAs in our microarray data, the related active enhancers identified in the LNCaP cells base in STARR-seq data (GSE82204) and identified in the PC-3 cells base in ENCODE data. There was no significant difference in the average signal between active eRNAs identified in ENCODE (average signal: 5.45) and all eRNAs (average signal: 5.54), but the average signal of active eRNAs identified in STARR-seq was 5.72, which was a little higher than all eRNAs.

STARR-seq is a massively parallel reporter assay to identify active enhancers based directly on their activity in entire genomes; the enhancers identified by STARR-seq may be in open or closed chromatin. As the work of Liu et al. showed, associated analysis of STARR-seq and DNaseI-seq data (ENCODE) from LNCaP cells found that only 11,980 (12.7%) of the 94,527 active enhancers were in open chromatin regions, and 82,547 active enhancers were in closed chromatin regions [14]. We also analyzed the expression of eRNAs transcribed from open and closed chromatin regions. Obviously, eRNAs that were transcribed from open chromatin (average signal: 6.25) exhibited increased expression compared with those from closed chromatin (average signal: 5.18) in LNCaP cells (Figure 2(a)).

### 3.3. Identification of Differentially Expressed SE-lncRNAs in Prostate Cancer

Superenhancers are clusters of enhancers that are formed by binding high levels of master transcription factors and mediator coactivators, which drive high levels of expression of genes that encode key regulators of cell identity [41]. dbSUPER is an integrated and interactive database of superenhancers [42]. To identify SE-lncRNAs contained in the microarray, we downloaded superenhancers from dbSUPER. All the identified superenhancers that overlapped lncRNAs contained in the microarray were further identified as SE-lncRNAs. A total of 14213 SE-lncRNAs were identified in the microarray; 292 differentially expressed SE-lncRNAs were then identified by Student's *t*-test using the threshold of *p* value < 0.05 and fold change ≥ 2 (Figure 2(c)). The distributions of differentially expressed SE-lncRNAs were analyzed, based on three criteria: classification, length, and chromosome locus (Figures 2(d)–2(f)).

To explore whether SE-lncRNAs had higher expression than eRNAs, we also analyzed the expression of all eRNAs and SE-lncRNAs. As expected, the average signal of SE-lncRNA (average signal: 5.90) was higher than that of eRNA in both tumor and corresponding adjacent normal tissues (*p* value ≤ 0.0001) (Figure 2(b)), although the differences were not very huge.

### 3.4. Potential Target Genes of the Differentially Expressed eRNAs Were Integrated with Differentially Expressed mRNAs

**Figure 3:**
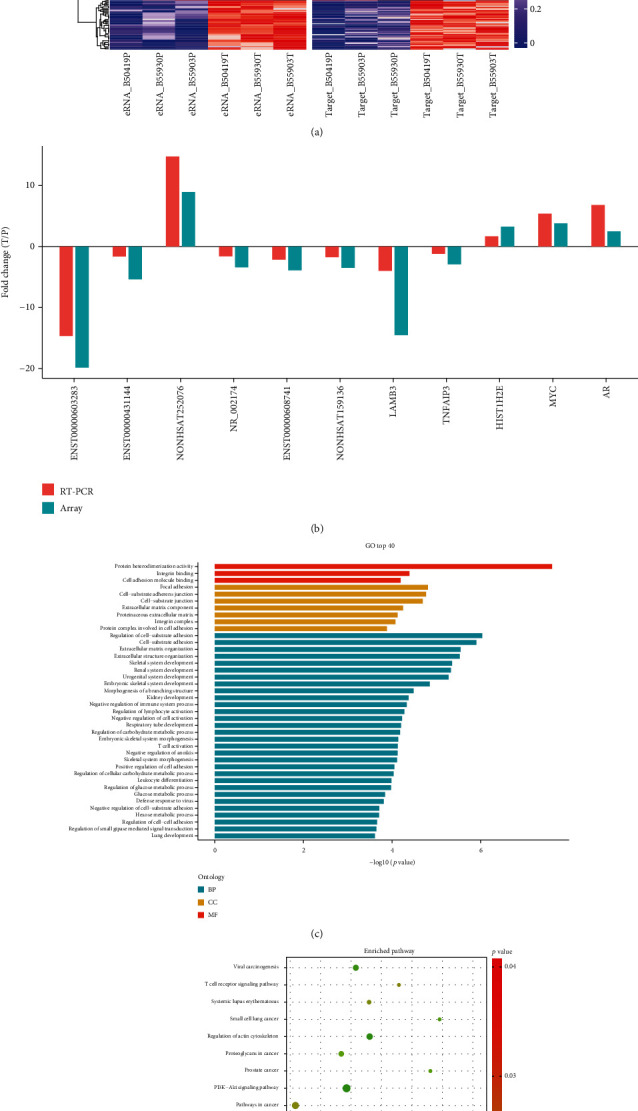
Bioinformatics analysis of target genes of differentially expressed eRNAs in a microarray and validation of selected genes by qRT-PCR. (a) Heat map that shows differentially expressed eRNAs and the positive differentially expressed target genes associated with them. (b) The barplot that shows the fold changes of RT-PCR and the microarray assay. The eRNA array data were validated by a RT-PCR assay. (c) Top 40 enriched gene ontology terms of target genes. (d) Top 20 enriched pathways of target genes. The prostate cancer-related pathway was enriched.

Although the role of most eRNAs in the regulation of gene expression remains unclear, it is considered a hallmark of functionally active enhancers. Other studies suggested that eRNAs mostly in cis to promote enhancer-promoter looping or recruit transcription factors (TFs) to specific enhancers [24, 28, 43–45]. The nearby genes of the differentially expressed eRNAs were defined as potential target genes. If an eRNA expressed differentially with its target gene concordantly, eRNA may play a regulatory role in its target gene expression. So, we integrated the target genes of eRNA with the mRNAs screened by the same microarray assay. A total of 325 differentially expressed target genes were identified for 295 differentially expressed eRNAs. Most of this eRNA (254, 86.1%) and related target genes (279, 85.8%) differentially expressed the same trend, which illustrated that eRNAs were associated with prostate cancer in the regulation of gene expression (Figure 3(a), Table 2, Supplementary Table 4).

### 3.5. Validation of the Differentially Expressed eRNAs with TCGA Data

To verify the microarray results, we downloaded RNA-seq data (*n* = 33) from TCGA (https://tcga-data.nci.nih.gov/tcga/) generated with nonrecurrent PCa tumors and normal tissues. Integrated analysis identified that 10144 eRNAs were in both the RNA-seq and the microarray data. A comparison of the differentially expressed genes in the microarray and RNA-seq showed that 267 differentially expressed eRNAs were identified in both the microarray and RNA-seq data (Supplementary Figure 1, Supplementary Table 2). One hundred and eleven eRNAs and their target genes were differentially expressed together, 95 of the 111 eRNAs had the same differentially expressed trend with the target gene, and the other 16 eRNAs were expressed at a low level in RNA-seq data.

### 3.6. Validation of the Microarray Data with qRT-PCR

To further verify the accuracy and reliability of the eRNA microarray results, six eRNAs and partial target genes and two PCa-associated genes (MYC and AR) were selected for a qRT-PCR assay. The housekeeping gene *β*-actin was used to normalize the expression level. The qRT-PCR results revealed that the 11 transcript level expressions were remarkably consistent with the microarray data (Figure 3(b)). This confirmed that the microarray data and the analysis based on these data were reliable and accurate, especially the prediction of the target genes of eRNAs. LAMB3, TNFAIP3, and HIST1H2BB were the target genes of ENST00000603283, ENST00000431144, and NONHSAT252076, respectively. LAMB3 was a gene known to be associated with prostate cancer in GeneCards, which is a well-known database that contains gene functions (https://www.genecards.org/).

### 3.7. Functional Enrichment Analysis of the Target Genes of eRNAs

To explore the functional implication of the target genes of eRNAs, we performed a functional enrichment analysis of the KEGG pathway using DAVID (https://david.ncifcrf.gov/) and GO by clusterProfiler, which are in the R package. A total of 20 KEGG pathways related to biological pathways were significantly enriched, and the results of the analysis revealed significant pathways were mainly related to prostate cancer (Table 3, Figure 3(d)). A total of 702 GO were significantly enriched, which included biological processes (*n* = 614), cellular components (*n* = 46), and molecular functions (*n* = 42) (Figure 3(c)). Differential expression of multiple target genes associated with inflammation, cellular adhesion, and apoptotic was observed.

We further analyzed the target genes of eRNAs using the GeneAnalytics tool of GeneCards (https://www.genecards.org/). Seventeen target genes were known to be associated with PCa (Table 4), 35 target genes were house-keeping genes, and 31 target genes were abundant genes in multiple organizations. The 17 genes associated with PCa were mainly involved in cancer and prostate cancer-related pathways, which include the prostate cancer pathway, focal adhesion pathway, PI3K-Akt signaling pathway, regulation of actin cytoskeleton pathway, ECM-receptor interaction pathway, proteoglycans in cancer pathway, pathways in cancer, and AMPK signaling pathway. The 17 genes associated with eRNAs may play a role in a similar way *in vivo*.

### 3.8. Analysis and Construction of eRNAs That Were Involved in Transcriptional Regulation Networks

**Figure 4:**
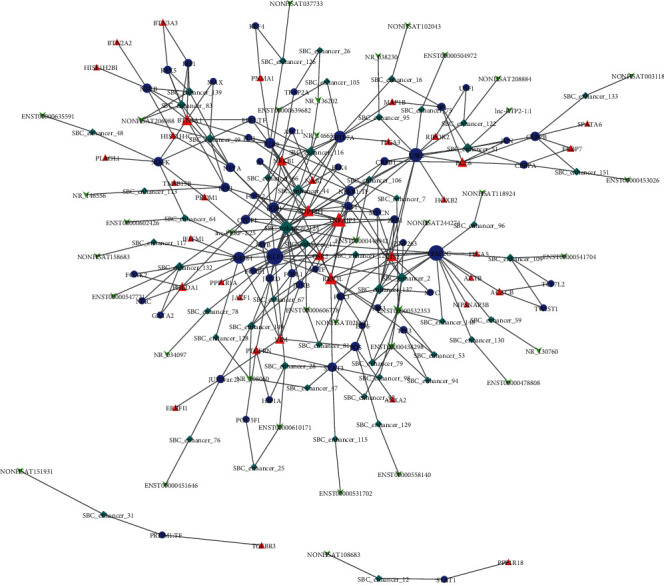
Construction of eRNA- (enhancer-) TF target gene coordination network. The network was generated using Cytoscape. The eRNAs (enhancers), TFs (transcription factors), and target genes are marked with squares (green), circles (purple), and triangles (red), respectively. The different sizes of nodes represent different degrees.

eRNAs are required for transcriptional activation, but their functional involvement is unclear. Previous studies suggested that eRNAs may promote enhancer-promoter looping or recruit transcription factors (TFs) to specific enhancers [31, 32]. Therefore, we investigated whether eRNAs and related enhancers were involved in transcriptional regulation. Motif analysis was done using HOMER (http://homer.ucsd.edu/homer/) on the promoters of target genes and differentially expressed eRNA-related enhancers in a microarray to predict TF motifs. If the paired eRNA (enhancer) target gene shared the same TF-binding motifs, the eRNA may promote recruited TFs or enhancer-promoter looping. To confirm this, we downloaded the chromatin interaction region data generated with 3C, 4C, and 5C and a ChIA-PET assay from the DENdb database, and we integrated these with previous eRNA- (enhancer-) TF target gene results. With the integrated results, we constructed an eRNA involved in the transcriptional regulation network that was based on an eRNA- (enhancer-) TF target gene coordination (Figure 4). eRNA- (enhancer-) TF target gene coordinations that were in the top 2 degrees of enhancers are shown in Table 5. There were 34 eRNA- (enhancer-) TF target gene coordinations, which was 23% of all 147 coordinations. The eRNA-related enhancers with the most degrees were ENST00000448942 (gene name: WAKMAR2-203, from ENSEMBL) and lnc-PERP-2:25 (gene name: lnc-PERP-2, from LNCipedia), which had the same target gene TNFAIP3. The protein encoded by this gene was a zinc finger protein and ubiquitin-editing enzyme, which has been shown to inhibit NF-kappa B activation and TNF-mediated apoptosis. NR_146633 was the second important eRNA in the network, which was a noncoding transcript of SUPT3H; NR_146633 was longer than the coding transcripts. SUPT3H was involved in the C-MYC pathway and transcriptional misregulation in the cancer pathway. Perhaps, NR_146633 was expressed with the coding transcripts concordantly to regulate downstream genes.

### 3.9. CTCF May Mediate Chromatin Looping

CTCF is a highly conserved gene that displays close to 100% homology among mice, chickens, and humans [46]. CTCF is a well-known insulator protein that possesses enhancer blocking functions and barrier functions, but CTCF plays a critical role in mediating chromatin looping rather than functioning as an insulator [50]. It can act both as a transcriptional repressor and an activator. To find out whether CTCF contributed to mediating chromatin looping of enhancer-promoters, the CTCF ChIP-seq data for LNCaP cells were downloaded from ENCODE. We measured whether CTCF was enriched in both enhancers and promoters of 147 eRNA- (enhancer-) TF target gene coordinations that we analyzed previously. Not surprisingly, we found that CTCF enriched both enhancers and promoters in 30 eRNA- (enhancer-) TF target gene coordinations. CTCF mediated the chromatin looping of enhancer-promoters. The most important enhancers were K562_Enhancer_301676, K562_Enhancer_297273, K562_Enhancer_288447, and K562_Enhancer_281124; the most important TFs were KLF5, TFAP2C, USF2, ZNF384, and EGR1.

### 3.10. eRNAs Regulated Androgen Response Genes and Further Affected Prognosis

Androgens and the androgen receptor (AR) play significant roles in the growth and progression of PCa. Androgens exert their biological and physiological effects by activating AR transcriptional activity [28]. Here, we investigated the expression of Hallmark “androgen response” genes and their potential regulated eRNAs between tumors and corresponding adjacent normal prostate tissues in a microarray. Fourteen eRNAs and the related “androgen response” genes were expressed differentially. For instance, eRNA ENSG00000225339, which has SPDEF (SAM Pointed Domain Containing ETS Transcription Factor) as a target gene, functions as an androgen-independent transactivator of prostate-specific antigen (PSA) promoters. Its related pathway is regulation of androgen receptor activity. Low expression of UBE2J1 is correlated with poor survival of PCa patients in TCGA data. Overall, these “androgen response” genes and their potential regulated eRNAs may be suitable targets for prognostic biomarkers or for developing therapies.

## 4. Discussion

Here, we screened the expression of eRNAs and SE-lncRNAs in three paired prostate tumor and corresponding adjacent normal prostate tissue samples using a microarray, and we identified 681 prostate cancer-associated differentially expressed eRNAs and 292 SE-lncRNAs. The nearby genes that were differentially expressed with related eRNAs concordantly were defined as target genes of eRNA. A total of 325 target genes for 295 differentially expressed eRNAs were identified. The target genes mostly participated in cancer occurrence and development-related pathways, which included prostate cancer, focal adhesion, PI3K-Akt signaling pathway, ECM-receptor interaction, viral carcinogenesis, proteoglycans in cancer, pathways in cancer, adherens junction, and AMPK signaling pathway. GO enrichment analysis revealed target genes that were mainly associated with inflammation, cellular adhesion, and apoptotic. The results of GeneAnalytics analysis produced 17 target genes that were known to be associated with PCa. In our microarray data, AR, MYC, and lncRNA PCA3 were upregulated, and PTEN was downregulated. This and qRT-PCR results confirmed that the microarray data and prediction of target genes were reliable and accurate. ENST00000603283, ENST00000431144, and NONHSAT252076 and their target genes LAMB3, TNFAIP3, and HIST1H2BB, respectively, and the known PCa-associated genes AR and MYC were verified by RT-PCR.

Furthermore, we described an eRNA involved in the transcriptional regulation network based on eRNA- (enhancer-) TF target gene coordination, and we found 147 eRNA- (enhancer-) TF target gene coordinations. In the coordination, enhancers and promoters of target genes shared the same TF-binding motifs, and the chromatin interaction of enhancers and promoters was proved by 3C, 4C, and 5C and a ChIA-PET assay. Also, the CTCF ChIP-seq data revealed that CTCF may be involved in eRNA- (enhancer-) TF target gene regulation networks by mediating chromatin looping; we found 30 potential CTCF-mediated eRNA- (enhancer-) TF target gene coordinations. eRNAs in the coordination promoted the enhancer-promoter looping or recruit transcription factors (TFs) to specific enhancers. We analyzed and constructed the eRNA involved transcriptional regulatory network using gene expression profiling data, transcription factor binding data, and chromosome interaction data.

Prostate cancer is initially androgen-dependent, but the effectiveness of androgen receptor (AR) inhibitors in recurrent disease is variable. The study of androgen-dependent prostate cancer transcriptional regulatory networks may help to elucidate the molecular mechanism of incipient prostate cancer and provide a foundation for the optimization of prostate cancer therapy strategies and development of drugs. The active eRNAs identified in LNCaP cells were positively correlated with our data of active eRNAs identified in PC-3 cells, mainly because the patients sampled for the microarray assay were androgen-dependent. LNCaP cells are androgen-sensitive human prostate adenocarcinoma cells, but PC-3 cells are derived from bone metastatic sites of prostate cancer patients that are recognized as androgen-independent. This result revealed that the function of eRNA and the transcriptional regulatory mechanism were different between androgen-dependent and androgen-independent prostate cancers. Our study was limited by relatively small sample size; so to validate the microarray results, we compared our data with the TCGA RNA-seq data generated with nonrecurrent PCa patients, and we verified 11 transcripts using qRT-PCR. We also found that eRNAs transcribed from open chromatin had increased expression compared with that transcribed from closed chromatin in LNCaP cells, which was consistent with the basic rules of transcription. Although the mechanisms of our eRNAs should be validated further, our results suggested a new perspective and clues for developing medicines and therapies for prostate cancer; for instance, ENSG00000225339 regulated a prognostic marker gene SPDEF positively that may also serve as a prognostic marker to promote more accurate prediction.

## 5. Conclusions

A comprehensive set of expressions of eRNAs and SE-lncRNAs was analyzed in PCa. The functions of eRNAs related to PCa were predicted by a series of bioinformatics analyses. These results furthered our understanding of PCa and should help to promote precision medicine.

## Figures and Tables

**Table 1:** The sources of eRNAs and differentially expressed eRNAs in a microarray.

	Total	DEN database	ENCODE	GSE82204
All eRNA sources	43680	40462	484	2734
Differentially expressed eRNA sources	681	658	3	20

**Table 2:** The top five up and down eRNAs differentially expressed the same trend with the target gene.

eRNA accession	*p* value	Fold change	Regulation	Target gene	*p* value	Fold change	Regulation
ENST00000624982	0.027	21.56	Down	IL10RA	0.046	2.76	Down
ENST00000458250	0.041	20.57	Down	HSD11B1	0.004	5.14	Down
ENST00000603283	0.020	19.88	Down	LAMB3	0.024	14.53	Down
lnc-SEL1L2-3:1	0.013	17.27	Down	FLRT3	0.008	17.09	Down
ENST00000504829	0.024	14.75	Down	SNCAIP	0.037	6.80	Down
NONHSAT252076	0.014	8.94	Up	HIST1H1T	0.037	8.79	Up
NONHSAT219894	0.008	6.12	Up	CORO2A	0.025	2.11	Up
NONHSAT175755	0.030	4.68	Up	SLC25A10	0.007	2.64	Up
NONHSAT056617	0.001	4.60	Up	FASN	0.022	5.44	Up
NONHSAT233757	0.025	4.40	Up	PLEKHH1	0.046	3.37	Up

**Table 3:** Enriched pathway of the target genes of differentially expressed eRNAs in a microarray.

Term	Pathway	Gene counts	*p* value	Enrichment
hsa04510	Focal adhesion	14	≤0.001	4.29
hsa04151	PI3K-Akt signaling pathway	16	≤0.001	2.93
hsa04810	Regulation of actin cytoskeleton	11	0.002	3.31
hsa04512	ECM-receptor interaction	7	0.002	5.08
hsa05203	Viral carcinogenesis	10	0.005	3.08
hsa05215	Prostate cancer	6	0.012	4.30
hsa05205	Proteoglycans in cancer	9	0.013	2.84
hsa05200	Pathways in cancer	13	0.020	2.09
hsa04520	Adherens junction	5	0.025	4.44
hsa04152	AMPK signaling pathway	6	0.044	3.08

**Table 4:** Seventeen known prostate cancer-associated target genes.

Target gene symbol	Target gene *p* value	Target gene fold change	Target gene regulation	eRNA accession	eRNA type
FASN	0.022	5.44	Up	NONHSAT056617	Exonic_sense
DCXR	0.005	3.85	Up	NONHSAT056617; NONHSAT238284	Exonic_sense; intergenic
PYCR1	0.028	3.16	Up	NONHSAT056617; NONHSAT175755	Exonic_sense; intergenic
HOXB13	0.017	2.43	Up	NONHSAT175319; NR_110330	Exonic_antisense; exonic_antisense
LCN2	0.036	17.45	Down	NONHSAT134945	Intronic_sense
LAMB3	0.024	14.53	Down	ENST00000458250; ENST00000603283	Intergenic; intergenic
IGF2	0.027	5.07	Down	NR_028043; NONHSAT158742	Exonic_antisense; exonic_antisense
RIPOR2	0.014	4.74	Down	NONHSAT208884; NR_002174; NONHSAT208894	Exonic_sense; intergenic; intergenic
BCL2	0.008	4.62	Down	ENST00000587475; NONHSAT178558	Intronic_sense; intronic_sense
STOM	0.015	4.02	Down	NR_003191	Intergenic
LY75	0.026	3.76	Down	NONHSAT242679	Intronic_sense
ALOX5AP	0.041	3.69	Down	NR_148425; NONHSAT167432	Exonic_sense; intergenic
FGFR1	0.012	3.33	Down	ENST00000519764; NR_148052	Intergenic; exonic_sense
FOXO1	0.018	3.05	Down	NONHSAT165576	Exonic_antisense
PIK3R1	0.001	2.99	Down	lnc-SLC30A5-6:1	Exonic_sense
COL4A1	0.019	2.87	Down	NONHSAT167267	Exonic_sense
MMP14	0.014	2.53	Down	NONHSAT234087	Exonic_antisense

**Table 5:** The top 2 degrees of enhancer in the network.

Enhancer accession	TF	eRNA accession	Target gene
K562_enhancer_301676	FOSL2	ENST00000448942	TNFAIP3
K562_enhancer_301676	USF2	ENST00000448942	TNFAIP3
K562_enhancer_301676	KLF5	ENST00000448942	TNFAIP3
K562_enhancer_301676	ZEB1	ENST00000448942	TNFAIP3
K562_enhancer_301676	EGR1	ENST00000448942	TNFAIP3
K562_enhancer_301676	JUND	ENST00000448942	TNFAIP3
K562_enhancer_301676	JUNB	ENST00000448942	TNFAIP3
K562_enhancer_301676	MITF	ENST00000448942	TNFAIP3
K562_enhancer_301676	CTCFL	ENST00000448942	TNFAIP3
K562_enhancer_301676	FOSL1	ENST00000448942	TNFAIP3
K562_enhancer_301676	EBF1	ENST00000448942	TNFAIP3
K562_enhancer_301676	CREB1	ENST00000448942	TNFAIP3
K562_enhancer_301676	MITF	lnc-PERP-2:25	TNFAIP3
K562_enhancer_301676	JUND	lnc-PERP-2:25	TNFAIP3
K562_enhancer_301676	NFKB1	lnc-PERP-2:25	TNFAIP3
K562_enhancer_301676	KLF5	lnc-PERP-2:25	TNFAIP3
K562_enhancer_301676	ZEB1	lnc-PERP-2:25	TNFAIP3
K562_enhancer_301676	FOSL2	lnc-PERP-2:25	TNFAIP3
K562_enhancer_301676	EGR1	lnc-PERP-2:25	TNFAIP3
K562_enhancer_301676	USF2	lnc-PERP-2:25	TNFAIP3
K562_enhancer_301676	FOSL1	lnc-PERP-2:25	TNFAIP3
K562_enhancer_301676	CTCFL	lnc-PERP-2:25	TNFAIP3
K562_enhancer_301676	EBF1	lnc-PERP-2:25	TNFAIP3
K562_enhancer_301676	CREB1	lnc-PERP-2:25	TNFAIP3
K562_enhancer_301676	JUNB	lnc-PERP-2:25	TNFAIP3
K562_enhancer_288447	NRF1	NR_146633	SUPT3H
K562_enhancer_288447	ELK4	NR_146633	SUPT3H
K562_enhancer_288447	AR	NR_146633	SUPT3H
K562_enhancer_288447	EGR1	NR_146633	SUPT3H
K562_enhancer_288447	ZNF384	NR_146633	SUPT3H
K562_enhancer_288447	MYCN	NR_146633	SUPT3H
K562_enhancer_288447	ZBTB7A	NR_146633	SUPT3H
K562_enhancer_288447	KLF9	NR_146633	SUPT3H
K562_enhancer_288447	KLF5	NR_146633	SUPT3H

## Data Availability

The data used to support the findings of this study are available in the GEO database (GSE155056) or from the corresponding author upon request.

## Abbreviations

PCa: Prostate cancer
lncRNA: Long noncoding RNA
eRNA: Enhancer RNA
SE-lncRNA: Superenhancer lncRNA
SE: Superenhancers
CTCF: CCCTC-binding factor
TF: Transcription factor
ENCODE: Encyclopedia of DNA elements
DHS: DNase I hypersensitivity sites
STARR-seq: Self-transcribing active regulatory region sequencing
p53BER: p53-bound enhancer regions
ER: Estrogen receptor
AR: Androgen receptor
NCBI: National Center for Biotechnology Information
GO: Gene Ontology
KEGG: Kyoto Encyclopedia of Genes and Genomes
DAVID: Database for Annotation Visualization and Integrated Discovery
TCGA: The Cancer Genome Atlas
RPKM: Reads per kilobase per million mapped reads
PSA: Prostate-specific antigen.

## Supplementary Materials

Supplementary Materials Supplementary Figure 1. Comparison of the differentially expressed eRNAs in the microarray and RNA-seq from TCGA. Venn diagram showing the overlap of the differentially expressed eRNAs identified by the microarray generated in our lab and RNA-seq from TCGA. Supplementary Table 1. All the primer sequences used in qRT-PCR assay. Supplementary Table 2. The fold changes and *p* values of differentially expressed eRNAs in RNA-seq data downloaded from TCGA and microarray data generated in our lab. List of differentially expressed eRNAs in microarrays and TCGA (from RNA-seq). Supplementary Table 3. The raw data of qRT-PCR. Supplementary Table 4. The expression of differentially expressed eRNAs and the same trend differentially expressed target genes and the expression of differentially expressed SE-lncRNAs and the same trend differentially expressed target genes in the microarray data.
