# Supplementary figures and images for: eRNAs and Superenhancer lncRNAs Are Functional in Human Prostate Cancer
Source: Dis Markers. 2020 Sep 22;2020:8847986. doi: 10.1155/2020/8847986 (PMC7532396; doi:10.1155/2020/8847986)

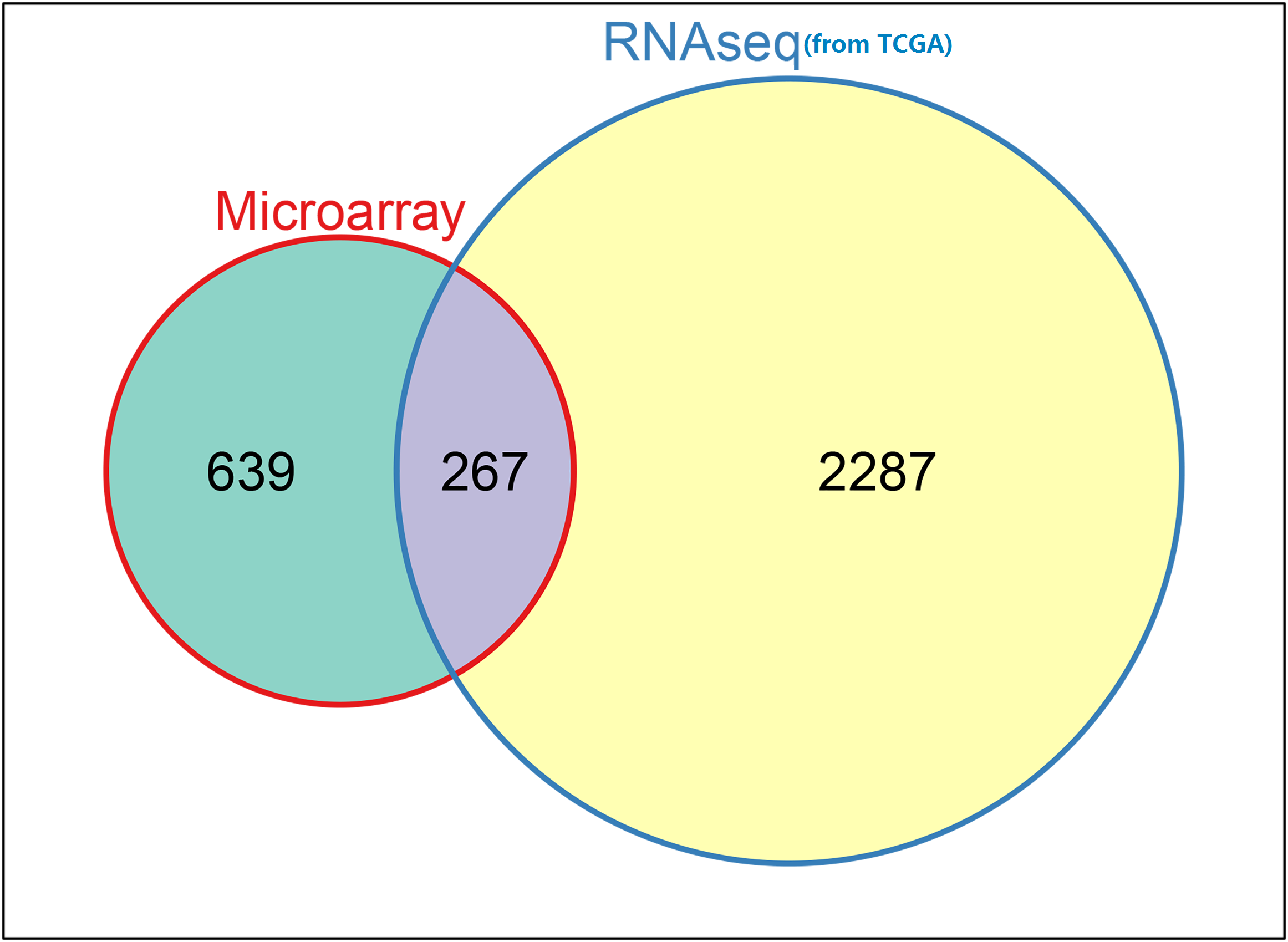

Supplement: Supplementary Materials — Supplementary Figure 1. Comparison of the differentially expressed eRNAs in the microarray and RNA-seq from TCGA. Venn diagram showing the overlap of the differentially expressed eRNAs identified by the microarray generated in our lab and RNA-seq from TCGA. Supplementary Table 1. All the primer sequences used in qRT-PCR assay. Supplementary Table 2. The fold changes and p values of differentially expressed eRNAs in RNA-seq data downloaded from TCGA and microarray data generated in our lab. List of differentially expressed eRNAs in microarrays and TCGA (from RNA-seq). Supplementary Table 3. The raw data of qRT-PCR. Supplementary Table 4. The expression of differentially expressed eRNAs and the same trend differentially expressed target genes and the expression of differentially expressed SE-lncRNAs and the same trend differentially expressed target genes in the microarray data. [file 8847986.f1.zip › Supplementary Figure 1 (1).png]
